# Hedgehog-GLI signalling promotes chemoresistance through the regulation of ABC transporters in colorectal cancer cells

**DOI:** 10.1038/s41598-020-70871-9

**Published:** 2020-08-19

**Authors:** Agnese Po, Anna Citarella, Giuseppina Catanzaro, Zein Mersini Besharat, Sofia Trocchianesi, Francesca Gianno, Claudia Sabato, Marta Moretti, Enrico De Smaele, Alessandra Vacca, Micol Eleonora Fiori, Elisabetta Ferretti

**Affiliations:** 1 Department of Molecular Medicine, Sapienza University of Rome, Viale Regina Elena 291, 00161 Rome, Italy; 2 Department of Experimental Medicine, Sapienza University of Rome, Viale Regina Elena 324, 00161 Rome, Italy; 3 Department of Oncology and Molecular Medicine, Istituto Superiore di Sanità, 00161 Rome, Italy; 4 Istituto Pasteur Italia - Fondazione Cenci Bolognetti, Viale Regina Elena 291, 00161 Rome, Italy

**Keywords:** Colorectal cancer, Cancer therapy, Cancer therapeutic resistance

## Abstract

Colorectal cancer (CRC) is a leading cause of cancer death. Chemoresistance is a pivotal feature of cancer cells leading to treatment failure and ATP-binding cassette (ABC) transporters are responsible for the efflux of several molecules, including anticancer drugs. The Hedgehog-GLI (HH-GLI) pathway is a major signalling in CRC, however its role in chemoresistance has not been fully elucidated. Here we show that the HH-GLI pathway favours resistance to 5-fluorouracil and Oxaliplatin in CRC cells. We identified potential GLI1 binding sites in the promoter region of six ABC transporters, namely ABCA2, ABCB1, ABCB4, ABCB7, ABCC2 and ABCG1. Next, we investigated the binding of GLI1 using chromatin immunoprecipitation experiments and we demonstrate that GLI1 transcriptionally regulates the identified ABC transporters. We show that chemoresistant cells express high levels of GLI1 and of the ABC transporters and that GLI1 inhibition disrupts the transporters up-regulation. Moreover, we report that human CRC tumours express high levels of the ABCG1 transporter and that its expression correlates with worse patients’ prognosis. This study identifies a new mechanism where HH-GLI signalling regulates CRC chemoresistance features. Our results indicate that the inhibition of Gli1 regulates the ABC transporters expression and therefore should be considered as a therapeutic option in chemoresistant patients.

## Introduction

Colorectal cancer (CRC) is a leading cause of cancer-related death worldwide and is characterized by resistance mechanisms that lead to disease progression^1^. Resistance can be achieved by the emergence of clones resistant to specific targeted drugs, and/or by the up-regulation of pathways involved in the detoxification of cells^2^.

ATP-binding cassette (ABC) transporters are a superfamily of genes encoding transmembrane proteins involved in the transport of several types of substrates irrespective of the concentration gradient, using the energy of the hydrolysis of ATP^3^. Forty-eight ABC transporters have been characterized in human, belonging to seven subfamilies (A to G). ABC transporters are heterogeneous regarding the type of substrate (hormones, lipids, ions, xenobiotics, etc.) and the specificity, since some are highly specific while others can transport a wide range of substrate^3^. Of note, anticancer drugs have been shown to be the substrate of numerous ABC transporters^4^ and the inhibition of certain ABC transporters may enhance the absorption of cytotoxic drugs^3^.

The Hedgehog (HH)–GLI signalling is a developmental pathway, conserved from flies to mammals, with central roles in development and homeostasis^5^. At the cellular level, the HH-GLI pathway is involved in the control of proliferation, differentiation, survival, tissue polarity and stem cell maintenance^6^. The canonical HH-GLI pathway is composed of secreted ligands (Sonic Hedgehog—SHH, Desert Hedgehog—DHH and Indian Hedgehog—IHH) that bind to and inactivate the transmembrane receptor Patched (PTCH), which in turn relieves its repression on a second transmembrane receptor Smoothened (SMO)^5^. This activation triggers intracellular molecular events that end up with the activation of the transcription factor GLI1. The disruption of regulatory mechanisms in the HH-GLI pathway is linked to tumorigenesis, tumour maintenance and cancer stem cell phenotype^5^. Of note, GLI1 can also be activated by non-canonical intracellular signalling^5^, referred as “oncogenic load”, such as Neuropilin2 in non-small cell lung cancer^7^ and KRAS in pancreatic ductal adenocarcinoma^8^.

HH-GLI signalling has been described as a major signalling in CRC maintenance and it was recently shown that it mediates anticancer drug resistance in patient-derived organoid cultures^9^. According to this, the present study aimed at analysing the role of the HH-GLI signalling in colorectal cancer (CRC) chemoresistance. Indeed, ABCG2, one of the most intensely studied ABC transporters, is a GLI1 transcriptional target and has been linked to HH-GLI1 dependant drug sensitivity^7, 10, 11^.

The above-described findings prompted us to investigate whether HH-GLI1 contributes to chemoresistance by regulating the expression of ABC transporters.

## Results

### HH-GLI sustains cell growth and GLI inhibition sensitizes CRC cells to chemotherapy

We aimed at understanding the role of the HH-GLI signalling in colorectal cancer (CRC) chemoresistance. To this end we first investigated the role of the HH-GLI signalling in the apoptosis and cell growth of CRC cells Colo205 treated with 5-fluorouracil (5-FU) and Oxaliplatin, cytotoxic drugs used in the treatment of advanced CRC^12^.

**Figure 1:**
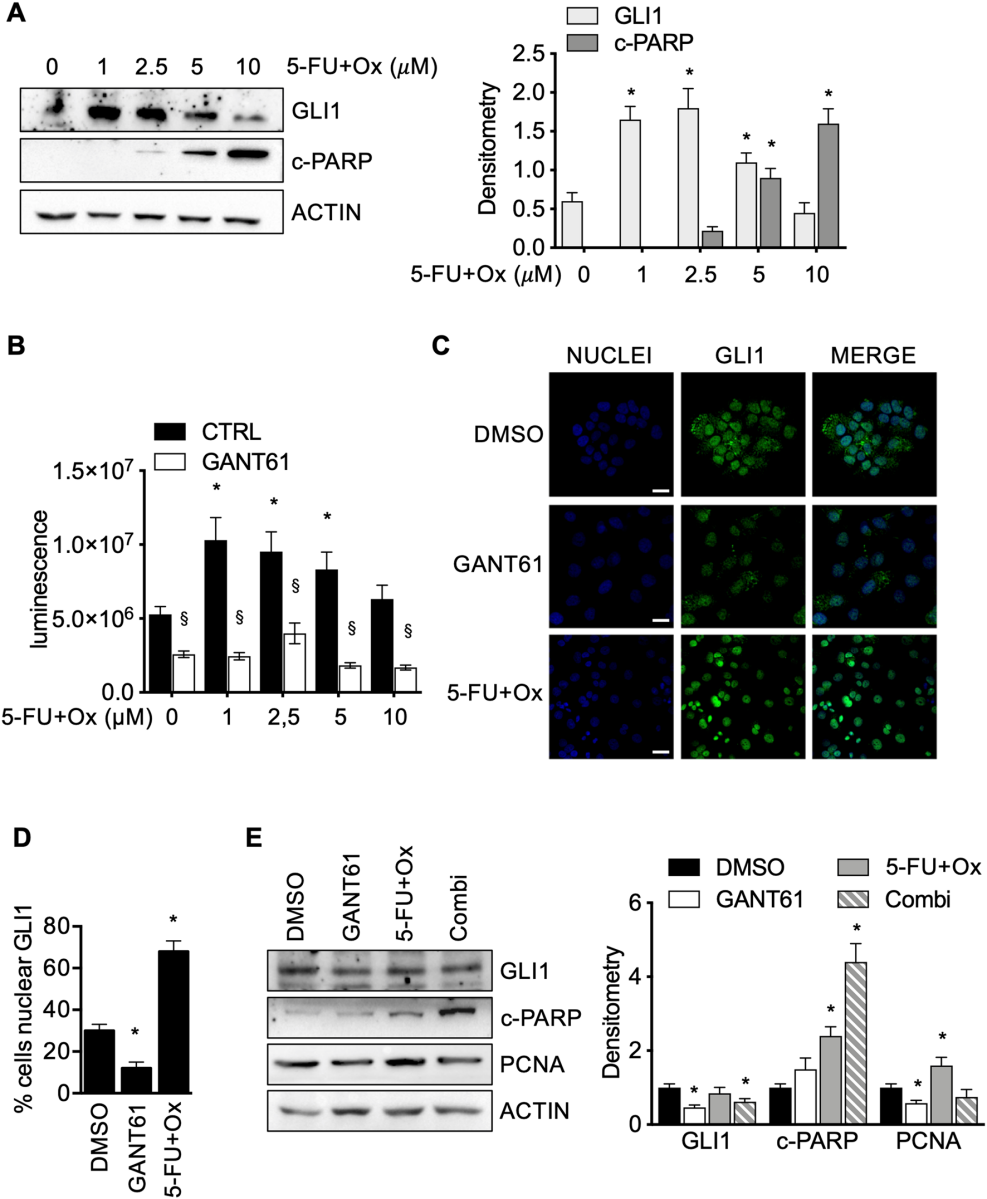
**(A)** Left: Western blot of endogenous GLI1 and cleaved PARP (c-PARP) in Colo205 cells treated with increasing doses of 5-FU and Oxaliplatin (5-FU + Ox) for 48 h. Loading control: ACTIN. Images are representative of three independent experiments; right: bar graphs show densitometrically quantified band intensity values normalized to the loading control. Data are representative of three independent experiments, *p < 0.05 versus control (two-way ANOVA test). Uncropped images are shown in Supplementary Fig. 5A. **(B)** Evaluation of cell viability, measured with Celltiter-GLO, of Colo205 cells treated with 5-FU + Ox in presence or absence of 10 μM GANT61 for 96 h. Data are representative of three independent experiments, *p  < 0.05 versus untreated; ^§^p < 0.05 5-FU + Ox versus GANT61 + 5-FU + Ox (two-way ANOVA test). **(C)** Immunofluorescence staining of GLI1 (green) in Colo205 cells treated with 10 μM GANT61 or 10 μM 5-FU + Ox for 48 h, as depicted in figure; Nuclei are counterstained with Hoechst. Bars, 20 μm. Images are representative of three independent experiments. Imaris 8.1 software (Oxford Instruments, https://imaris.oxinst.com/) was used for image-processing. **(D)** Histogram showing the percentage of cells displaying nuclear GLI1. The experiment was performed in triplicate, *p < 0.05 (Mann–Whitney U test). **(E)** Left: representative western blot of endogenous GLI1, cleaved PARP (c-PARP) and PCNA in Colo205 cells treated with 10 μM 5-FU + Ox ,10 μM GANT61 or both (Combi) for 48 h. Loading control: ACTIN. Right: Bar graphs show densitometrically quantified band intensity values normalized to the loading control. Data are representative of three independent experiments, *p < 0.05 versus control (two-way ANOVA test). Uncropped images are shown in Supplementary Fig. 5B. Histograms were created using GraphPad Prism version 6.0 for macOS, https://ww.graphpad.com.

5-FU and Oxaliplatin treatment of Colo205 cells for 48 h resulted in the up-regulation of GLI1 protein expression with a dose of up to 5 μM, while this was not evident at 10 μM treatment (Fig. 1A). 5-FU and Oxaliplatin treatment at 5 and 10 μM was also accompanied by a significant increase in apoptosis, measured by cleaved PARP (c-PARP) (Fig. 1A).

Coherently, Colo205 showed significantly enhanced cell viability when exposed to up to 5 μM of 5-FU and Oxaliplatin compared to untreated cells (Fig. 1B). However, if cells were previously exposed to the GLI inhibitor GANT61^13^, cell viability was significantly impaired, indicating that GLI inhibition can overcome the growth-promoting effects of a low dose of 5-FU and Oxaliplatin (Fig. 1B). Since 5-FU and Oxaliplatin treatment resulted in GLI1 expression induction, we then proceeded to investigate the subcellular localization of the transcription factor GLI1 in Colo205 treated with 5 μM of 5-FU and Oxaliplatin. While in untreated cells GLI1 was localized both in the cytoplasm and in the nucleus (Fig. 1C), in treated cells GLI1 staining was essentially localized in the nucleus with significantly more cells displaying nuclear GLI1 (Fig. 1D), an observation coherent with an enhanced GLI1 transcriptional activity. GANT61 treated cells showed a weaker staining of GLI1 protein with fewer cells showing nuclear GLI1 (Fig. 1C, D). We then investigated whether the effects of 5-FU plus Oxaliplatin and GANT61 on Colo205 growth could be attributable to modulation in cell proliferation or apoptosis. Proliferation, measured through PCNA protein levels, was impaired by GANT61 treatment and enhanced by 5-FU plus Oxaliplatin treatment (Fig. 1E). Pre-treating cells with GANT61 prevented the proliferative effect of 5-FU plus Oxaliplatin on cells (Fig. 1E). Interestingly, we observed that apoptosis was slightly albeit significantly affected by 5-FU plus Oxaliplatin treatment and the combination with GANT61 was able to significantly increase this effect (Fig. 1E).

### ABC transporters are transcriptionally regulated by HH-GLI

Since drug resistance could be associated with modified expression of ABC transporters, we wondered whether HH-GLI signalling was involved in their regulation.

**Figure 2:**
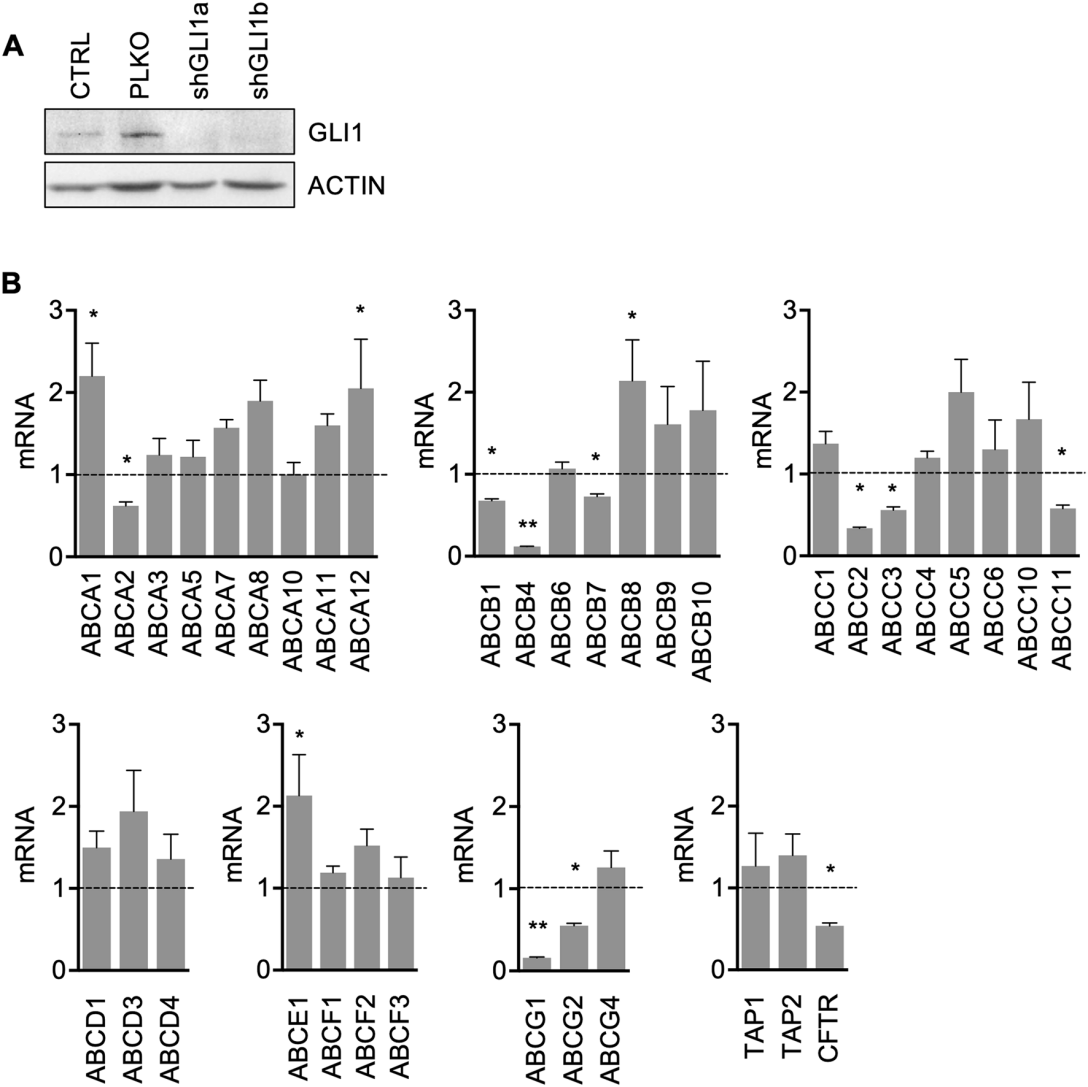
**(A)** Western blot of endogenous GLI1 in Colo205 at basal level, after short hairpin mediated GLI1 silencing using two different short hairpin clones (shGLI1a and shGLI1b) and non targeting control (PLKO) after 72 h from infection. Loading control: ACTIN. Images are representative of at least three independent experiments. Uncropped images are shown in Supplementary Fig. 5C. **(B)** Histograms showing mRNA levels expressed in arbitrary units of ABC transporters expressed by Colo205 cells after short hairpin mediated GLI1 silencing (shGLI1) after 72 h from infection. Dashed line: non targeting control (PLKO). *p < 0.05 versus PLKO; **p < 0.01 versus PLKO (one-way ANOVA test). Data are means ± SD from three independent experiments. Histograms were created using GraphPad Prism version 6.0 for macOS, https://www.graphpad.com.

To this end, we performed a qPCR-based array of all ABC transporters in Colo205 after short hairpin mediated GLI1 silencing (shGLI1) (Fig. 2A). ABCG2 was already demonstrated to be a transcriptional target of GLI1^11^, so we used it as a positive control in our experiments.

We found that thirty-six ABC transporters were expressed by Colo205 cells and are reported, according to subfamilies, in Fig. 2B. Interestingly, 14 ABC transporters were significantly modulated by GLI1 silencing; in detail 10 of them were down-regulated (ABCA2, ABCB1, ABCB4, ABCB7, ABCC2, ABCC3, ABCC11, ABCG1, ABCG2 and CFTR) and 4 were up-regulated (ABCA1, ABCA12, ABCB8 and ABCE1) (Fig. 2B). Since our aim was the identification of ABC transporters transcriptionally regulated by HH-GLI1 signalling, we chose to focus on those resulting down-regulated after GLI1 inhibition.

**Figure 3:**
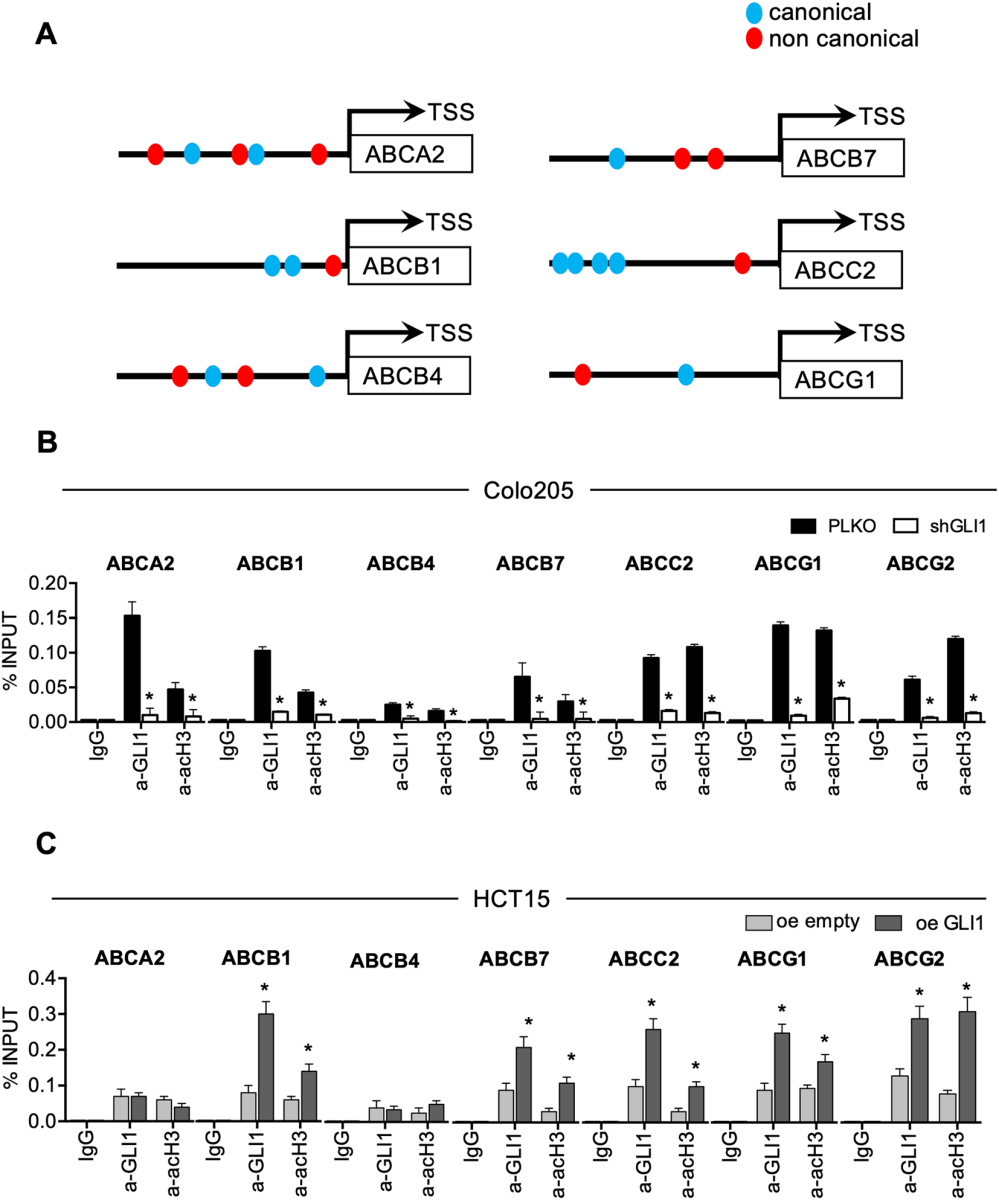
**(A)** Schematic representation of the promoter region of the indicated ABC transporters showing locations of putative GLI1 canonical and non canonical binding sites; see supplementary Fig. 1 for detailed sequences. **(B)** qPCR-ChIP assay of endogenous GLI1 occupancy of the promoter region of the indicated ABC transporters in Colo205 cells after short hairpin mediated GLI1 silencing (shGLI1) versus non targeting control (PLKO) for 72 h. Immunoprecipitation with IgG was performed as control. Anti-acetyl-H3 (a-acH3) antibody was used to detect ABC transporters transcriptional activation. *p < 0.05 versus PLKO (Mann–Whitney U test). **(C)** qPCR-ChIP assay of overexpressed GLI1 occupancy of the promoter region of the indicated ABC transporters in HCT15 cells after GLI1 overexpression (oe GLI1) or control (oe empty) for 24 h. Immunoprecipitation with IgG was performed as control. Anti-acetyl-H3 (a-acH3) antibody was used to detect ABC transporters transcriptional activation. *p < 0.05 versus oe empty; **p < 0.01 versus oe empty (Mann–Whitney U test). **(B, C)** Data are means ± SD from at least 3 independent experiments. Histograms were created using GraphPad Prism version 6.0 for macOS, https://www.graphpad.com.

To this end, we searched for GLI1 putative binding sites on the proximal promoter region of the ABC transporters. We analysed the region encompassing the 1,000 bases upstream the transcription starting sites (TSS) for the presence of canonical (GACCACCCA) and non canonical (CGCCTCCAG) GLI consensus sequences^14^ Indeed, we found GLI consensus sequences for six transporters (ABCA2, ABCB1, ABCB4, ABCB7, ABCC2 and ABCG1) (Fig. 3A and supplementary Fig. 1). The rationale behind the selection of the investigated ABC transporters is depicted in Supplementary Fig. 2.

Therefore, we performed chromatin immunoprecipitation (ChIP) experiments to investigate the recruitment of GLI1 protein on the promoter of the six selected ABC transporters.

We found recruitment of GLI1 on the promoter of all six ABC transporter analysed with a significant down-modulation in shGLI1 Colo205 cells (Fig. 3B); moreover, we observed a significant down-modulation of the transcriptional activation marker acetyl histone H3 recruitment on the ABC transporters promoters after silencing of GLI1 (Fig. 3B).

**Figure 4:**
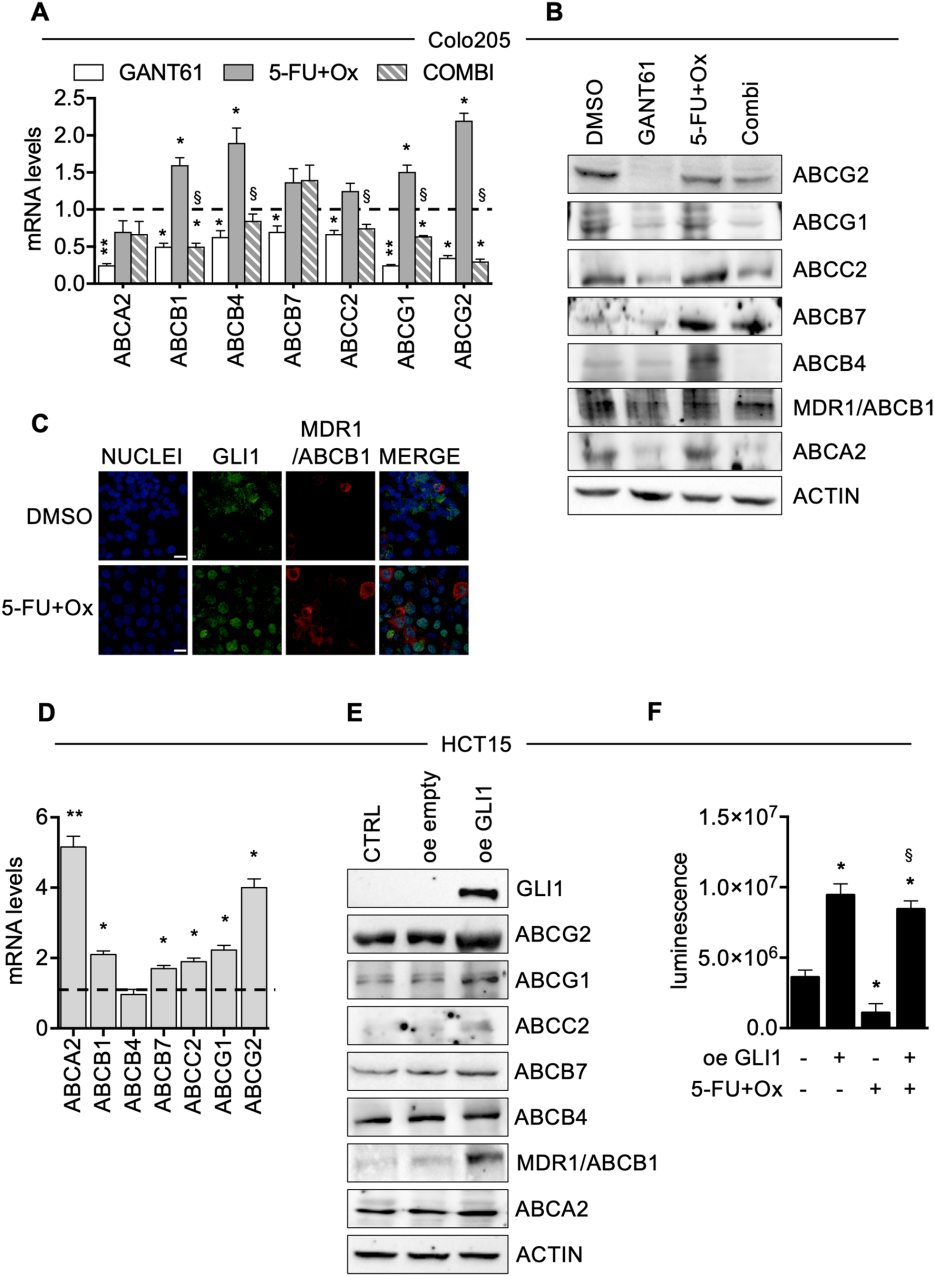
**(A)** Histograms showing mRNA levels of ABC transporters in Colo205 treated with 10 μM GANT61, 10 μM 5-FU and Oxaliplatin (5-FU + Ox) or the combination (combi) for 48 h. Data are representative of three independent experiments, *p < 0.05 versus Ctrl; § p < 0.05 GANT61 + 5-FU + Ox versus 5-FU + Ox (Two-way ANOVA test). **(B)** Western blot of endogenous ABC transporters in Colo205 cells treated with GANT61, 5-FU + Ox or the combination (combi). Loading control: ACTIN. Data are representative images from 3 independent experiments. Uncropped images are shown in Supplementary Fig. 6A. **(C)** Immunofluorescence staining of MDR1/ABCB1 (red) and GLI1 (green) in Colo205 cells treated with 10 μM 5-FU + Ox or control (DMSO) for 48 h. Nuclei are counterstained with Hoechst. Bars, 20 μm. Images are representative of at least three independent experiments. Histograms of MDR1/ABCB1 number of positive cells and intensity are shown in supplementary Fig. 3A. Imaris 8.1 software (Oxford Instruments, https://imaris.oxinst.com/) was used for image-processing. **(D)** Histograms showing mRNA levels of ABC transporters in HCT15 cells after GLI1 over expression (oe GLI1) for 24 h. Dashed line: Control transfected cells. Data are representative of three independent experiments, *p < 0.05 versus Ctrl; **p < 0.01 versus Ctrl (One-way ANOVA test). **(E)** Western blot of endogenous GLI1 and ABC transporters in HCT15 after GLI1 over expression (oe GLI1), transfection with empty vector (oe empty) and in non-transfected cells (CTRL) for 24 h. Loading control: ACTIN. Data are representative images from 3 independent experiments. Uncropped images are shown in Supplementary Fig. 6B. **(F)** Cell viability of HCT15 cells after overexpression of GLI1, after treatment with 10 μM 5-FU + Ox and after the combination of GLI1 overexpression and 5-FU + Ox. 5-FU + Ox treatment was added after 24 h from the transfection and it was carried out for 48 h. Data are representative of three independent experiments, *p  <  0.05 versus control; ^§^p < 0.05 GLI1 + 5-FU + Ox versus 5-FU + Ox (one-way ANOVA test). Histograms were created using GraphPad Prism version 6.0 for macOS, https://www.graphpad.com.

To validate our results, we overexpressed GLI1 in HCT15, a cell line characterized by low levels of GLI1, and we observed the recruitment of GLI1 on the promoters of ABCB1, ABCB7, ABCG1 and ABCG2, along with higher acetyl histone H3 recruitment (Fig. 3C). The following set of experiments was designed to validate our results. First of all, we analysed mRNA expression of the ABC transporters in Colo205 after drug-mediated GLI inhibition. Indeed, GANT-treated Colo205 cells showed decreased mRNA levels of all six ABC transporters analysed along with ABCG2 (Fig. 4A) and decreased protein levels of ABCA2, ABCB1, ABCC2, ABCG1 and ABCG2 (Fig. 4B). 5-FU and Oxaliplatin treatment caused an increase of mRNA levels of ABCB1, ABCB4, ABCG1 and ABCG2 (Fig. 4A) and of protein levels of ABCB4, ABCB7 and ABCC2 (Fig. 4B). Interestingly, when we combined short-term treatment of GANT61 and 5-FU plus Oxaliplatin, GANT61 treatment was able to overcome the inducing effects of 5-FU plus Oxaliplatin on some ABC transporters expression (Figs. 4A, B). Of note, immunofluorescence staining confirmed the increase of the number and intensity of ABCB1/MDR1 and of ABCG2 positive cells and showed co-localization with GLI1 in 5-FU plus Oxaliplatin treated cells (Fig. 4C and supplementary Fig. 3A–C).

Moreover, in HCT15 cells over-expressing GLI1 we observed increased mRNA levels of ABCA2, ABCB1, ABCB7, ABCC2, ABCG1 and ABCG2 (Fig. 4D) and an increase of protein levels of ABCA2, ABCB1, ABCB7, ABCG1 and ABCG2 (Fig. 4E).

Finally, GLI1 over-expression was able to increase cell growth in HCT15 cells, while 5-FU and Oxaliplatin treatment impaired cell growth (Fig. 4F); the combination of GLI1 over-expression and 5-FU and Oxaliplatin treatment resulted in an increase in cell proliferation (Fig. 4F) with respect to 5-FU and Oxaliplatin, therefore ectopic GLI1 was able to overcome 5-FU and Oxaliplatin effects on cell growth.

### Role of HH-GLI signalling in in-vitro induced chemoresistance

**Figure 5:**
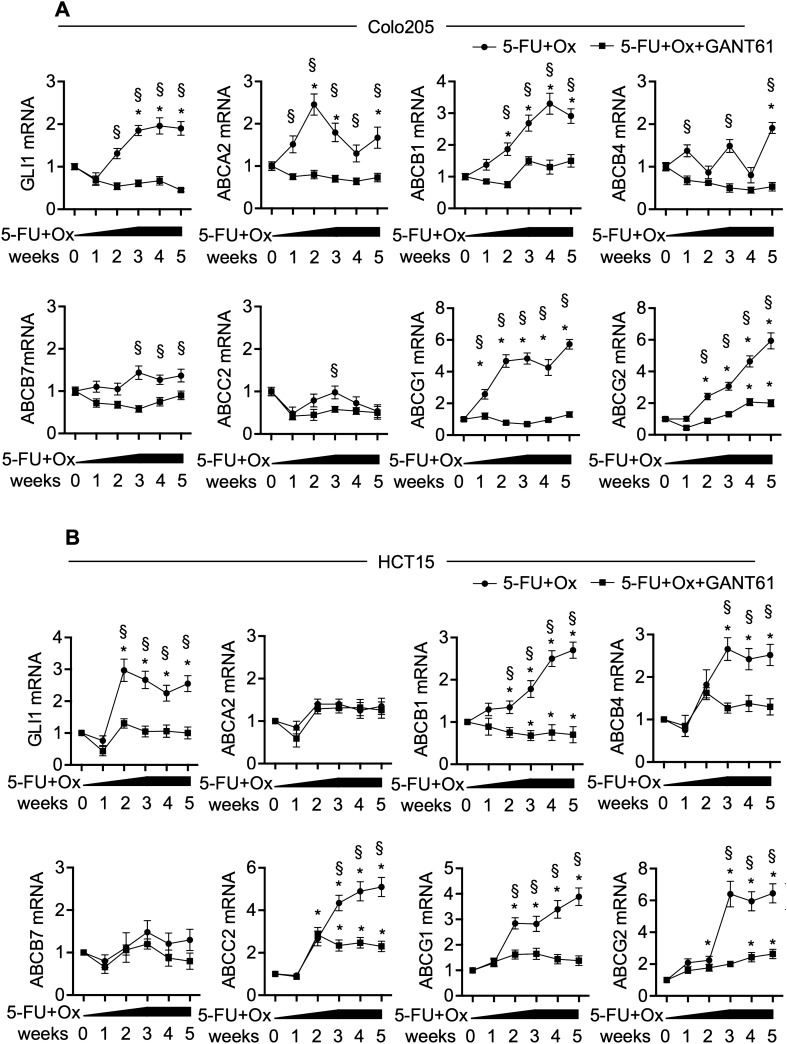
**(A)** mRNA levels of GLI1 and indicated genes, expressed in arbitrary units, in Colo205 treated with increasing doses of 5-FU and Oxaliplatin (5-FU + Ox), starting from 1 μM to 10 μM and 10 μM GANT61 during 5 weeks. *p < 0.05 versus starting values (two-way ANOVA test). **(B)** mRNA levels of GLI1 and indicated genes, expressed in arbitrary units, in HCT15 treated with increasing doses of 5-FU + Ox, starting from 1 to 10 μM and 10 μM GANT61 during 5 weeks. *p < 0.05 versus starting values; ^§^p < 0.05 5-FU + Ox versus 5-FU + Ox + GANT61 (two-way ANOVA test). Data are representative of three independent experiments, **(A, B)**. Histograms were created using GraphPad Prism version 6.0 for macOS, https://www.graphpad.com.

To further investigate the role of HH-GLI signalling in CRC chemoresistance, we induced chemoresistance in CRC cells by treating cells with 5-FU and Oxaliplatin, as described previously^15^, carrying out the treatment for 5 weeks (supplementary Fig. 4A, B). To verify the resistance, we compared the number of cells after 48 h of 5-FU and Oxaliplatin treatment in parental and resistant cells and we found that resistant cells were significantly more than parental cells, both in Colo205 and in HCT15 (supplementary Figs. 4C, 5D). We then analysed the expression of GLI1 and of the six GLI1-regulated ABC transporters in Colo205 and HCT15 cells during the induction of drug resistance (Fig. 5).

Expression of both GLI1 and of the ABC transporters ABCA2, ABCB1, ABCB4, ABCG1 and ABCG2 increased with drug treatment in Colo205 (Fig. 5A). In HCT15, we observed that the treatment up-regulated the expression of GLI1 and of the ABC transporters ABCB1, ABCB4, ABCC2, ABCG1 and ABCG2 (Fig. 5B). Interestingly, if we added GANT61 to the chronic 5-FU and Oxaliplatin treatment in order to prevent GLI1 up-regulation, ABC transporters failed to increase; ABCC2 in HCT15 and ABCG2 in both cell lines were still significantly up-regulated with respect to the starting population, however expression levels were significantly lower with respect to the relative 5-FU plus Oxaliplatin treated cells (Fig. 5A, B).

Taken together, our experiments demonstrated that six ABC transporters were regulated by the HH-GLI signalling, their expression levels were increased in a chemoresistance promoting condition, and this modulation was HH-GLI dependent. We observed that, among the 14 ABC transporters that were modulated by GLI1 silencing, not all resulted to be directly targeted by GLI1 in ChIP experiments, a plausible observation since HH-GLI can regulate their expression levels in an indirect fashion.

### In silico analyses of ABC transporters in CRC patients

To validate our results from CRC cellular models, we analysed GLI1 regulated ABC transporters in samples from CRC patients by re-interrogating publicly available CRC datasets using the R2 platform^16^. The following datasets were selected for our analyses: Watanabe^17^, that investigated gene expression in 53 samples of non-neoplastic rectal mucosa and 67 samples of CRC; Galamb^18^, that investigated gene expression in 6 microdissected samples from CRC and 6 microdissected samples from non-neoplastic tissues; Carmical^19^, that investigated gene expression in colorectal CD133+ cancer cells and in cancer-associated fibroblasts (CAFs).

Differential expression and p values of the analysed transporters are reported in Table 1.

**Table 1:** Modulation and statistical significance of the selected ABC transporters in the indicated datasets.

	Watanabe		Galamb		Carmical	
	Up/down in cancer vs n.c	p value	Up/down in cancer vs n.c	p value	Up/down in CD133 + vs CAFs	p value
ABCA2	Up	9.13e−51	ns	0.200	ns	0.09
ABCB1	Down	1.41e−08	Up	0.023	Down	6.26e−05
ABCB4	ns	0.926	ns	0.387	Down	0.017
ABCB7	Up	7.4e−07	ns	0.448	Up	0.032
ABCC2	Up	1.45e−18	ns	0.252	Up	1.08e−03
ABCG1	Up	7.70e−55	Up	0.029	Up	0.0002
ABCG2	Up	6.04e−03	ns	0.187	Up	0.042

**Figure 6:**
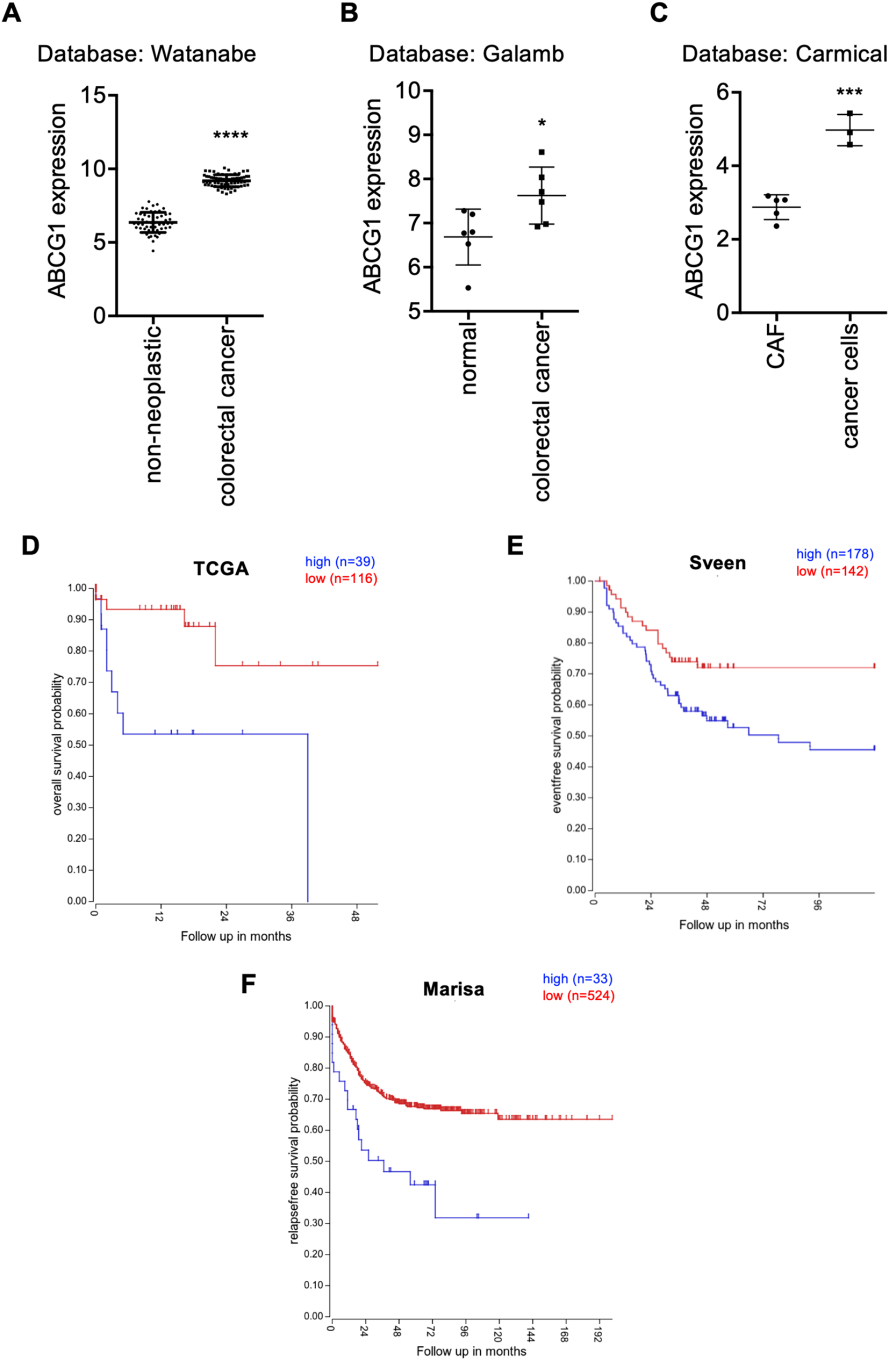
**(A–C)** Expression data of ABCG1 from datasets interrogated on R2 platform as indicated in main text. *p < 0.05; **p < 0.01, ****p < 0.0001 (Mann–Whitney U test). **(D–F)** Kaplan Meier curves of ABCG1 in the indicated datasets described in the main text. Histograms were created using GraphPad Prism version 6.0 for macOS, https://www.graphpad.com.

Among Gli1 regulated ABC transporters, ABCG1 was expressed at significantly higher levels in cancer tissues respect to non-tumour tissues (Fig. 6A, B) and in CD133+ cancer cells respect to CAFs (Fig. 6C).

Moreover, we investigated if there was any association between the expression levels of the ABC transporters of interest and the overall survival of patients. The following datasets were queried: TCGA mixed colon adenocarcinoma, that investigated overall survival probability in 174 samples; Tumor Colon (Core-Transcript) Sveen, that investigated event-free survival probability in 333 samples, Tumor Colon CIT (Combat) Marisa that investigated relapse-free survival probability in 566 samples and Tumor Colon MVRM—SieberSmith, that investigated relapse free survival probability in 345 samples. The results and statistical significance associated with expression levels of ABC transporters of interest are reported in Table 2.

**Table 2:** Survival data and statistical significance of the selected ABC transporters in the indicated datasets.

	TCGA		Sveen		Marisa		SieberSmith	
	High/low is worse	p value	High/low is worse	p value	High/low is worse	p value	High/low is worse	p value
ABCA2	Low	0.036	High	2.8e−06	Low	0.037	Low	4.3e−04
ABCB1	ns	0.082	Low	9.8e−05	High	0.023	High	1.5e−03
ABCB4	ns	0.083	ns	0.066	High	9.2e−03	High	5.1e−03
ABCB7	ns	0.084	Low	5.3e−03	ns	0.075	High	0.047
ABCC2	ns	0.156	High	0.014	Low	1.1e−03	Low	0.040
ABCG1	High	2.3e−03	High	3.4e−04	High	6.4e−04	Low	0.016
ABCG2	ns	0.085	High	2.5e−04	High	1.2e−03	Unknown	0.02

High expression levels of ABCG1 transporter were significantly associated with worse prognosis in three out of four datasets that we investigated, namely TCGA, Sveen and Marisa, as shown in the Kaplan Meier curves (Fig. 6D–F).

We observed that the dataset reporting discordant results (Sieber Smith) combines data from 2 smaller datasets, Sieber (290 samples) and Smith (55 samples), and the Kaplan Meier analysis of each dataset alone did not result in a significant association of ABCG1 and prognosis. Even though not statistically significant, the Smith dataset showed a concordant trend with those of TCGA, Sveen and Marisa. Unfortunately, we were not able to rule out that the difference in the Kaplan Meier trend could be ascribed to the characteristics of the Sieber cohort patients. Importantly, the three datasets showing significant association of high levels of ABCG1 and worse prognosis (TCGA, Sveen and Marisa) use different platforms and therefore different probes for the detection of ABCG1, and we believe that a concordance of results using different probes strengthens our results.

## Discussion

Despite recent advances in cancer therapy (i.e. screening programs allowing the surgical treatment of early-stage CRC patients and targeted therapies) CRC is still among the prevalent causes of cancer-related death^1^. Failure of therapeutic strategies is mainly linked to drug resistance that can be determined by an enhanced ability of cancer cells to detoxify by actively pumping cytotoxic drugs out of the cells, mediated by ABC transporters^3^. Interestingly, increased activity and expression of efflux pumps and detoxifying machinery has been detected in cancer stem cells, the subpopulation of cancer cells mainly responsible for drug resistance and recurrence^3, 20^.

The expression and role of ABC transporters in CRC have been intensely investigated, in vitro and in cohorts of patients. The expression of ABCB1 (MDR1/P-GP) has been previously reported as induced by chemotherapeutic agents in CRC cell lines^21^. ABCG2 was also associated with reduced toxicity and intracellular concentration of different types of chemotherapeutics^21^.

Signalling involved in CRC maintenance has been previously shown to regulate ABC transporters. Nuclear factor-kappaB (NF-κB) was shown to regulate ABCB1 and inhibition of NF-κB was able to sensitize colorectal cancer cells to chemotherapy in an ABCB1 dependent way^21^. ABCC3 was proved to be transcriptionally regulated by WNT signalling in CRC, thus likely contributing to acquired drug resistance^22^. Furthermore, MALAT1 silencing down-regulated the expression of ATP-binding cassette transporters (ABC), breast cancer resistance protein (BCRP), and multi-drug resistance proteins including MDR1 and MRP1, resulting in decreased resistance of CRC cells to 5-FU^23^.

Interestingly, given the wide overlap of the substrate, the potential of many ABC transporters involved in resistance to therapy is high. Moreover, the induction of multidrug resistance by treatment with a first drug could associate with the resistance to different chemotherapeutic agents, thus making therapy more difficult^21^. For example, CRC LoVo cells showed high levels of ABCG2, and consequently multidrug resistance, after oxaliplatin treatment and resulted resistant to 5‐FU and doxorubicin, too^24^.

ABC transporters have been associated with CRC risk and toxicity in different studies. On one hand, both ABCB1 and ABCG2 haplotypes were associated with risk of CRC in a Danish cohort^25^. On the other hand, ABC transporters polymorphisms were also correlated with toxic side effects caused to healthy cells. In detail in two independent cohorts, ABCC5 and ABCG1 haplotypes were associated with increased severe toxicity^26^. In a different study, SNPs in ABCB1 and ABCC4 were associated with a severe adverse reaction, including haematological toxicity in CRC patients^27^.

Given their undeniable role in conferring resistance to chemotherapy, ABC transporters have been investigated as therapeutic targets. Unfortunately, three generations of inhibitors of the most studied ABC transporters ABCB1 (P-gp) and ABCG2 did not meet expectations in terms of successful clinical trials, mostly due to unforeseen pharmacokinetic interactions, poor selectivity, low potency and high toxicity^21^. Interestingly, the role of ABC transporters in resistance is generally attributed to increased expression and not to acquired mutations^3^. This opens possibilities to indirect targeting of ABC transporters through direct targeting of active signalling pathways in CRC.

Interestingly, the HH-GLI pathway is involved in intense cross-talk with numerous signalling pathways that promote cancer progression, activating GLI in absence of the canonical PTCH/SMO pathway^5^. HH-GLI has been shown to regulate CRC cells maintenance, including cancer stem cell phenotype, metastasis and chemoresistance. Zhang et al.^28^ showed that GLI1 and GLI2 mediate 5-FU resistance in CRC cell line LoVo. GLI1 inhibition was shown to revert chemo-resistance in organoids from colorectal cancer patients, and concurrently down-regulated cancer stem cells markers, such as c-Myc, CD44 and Nanog^9^. Our results showed a direct involvement of Gli1 in chemoresistance of CRC cells. Indeed, we demonstrated that GLI1 modulates the expression level of a subset of ABC transporters, and through the modulation of GLI1 activity it is possible to impair chemoresistance in CRC cells.

Among the ABC transporters directly regulated by GLI1, ABCG1 is a good prognosis biomarker. Indeed, ABCG1 was expressed at high levels in CRC cells with respect to non-cancerous tissues and to CAFs, and its expression levels in primary tumours are associated with worse prognosis.

ABCG1 is a sterol transporter, thus contributing to cholesterol homeostasis in non-cancerous tissues^29^, however previous studies have described another role for ABCG1 in cancer. Indeed, ABCG1 was found to be involved in multidrug resistance in osteosarcoma, where it was up-regulated in cells with induced doxorubicin resistance^30^. Interestingly, this phenotype displayed also stem-like features, i.e. colony formation ability. Moreover, ABCG1 was recently reported to induce resistance to oxaliplatin and saracatinib and to be regulated by WNT signalling in hepatocellular carcinoma^31^.

Interestingly, SNPs correlating with higher levels of mRNA expression of ABCG1 were correlated with worse prognosis in non-small cell lung cancer^32^.

Of note, our results showed that 4 ABC transporters were up-regulated after GLI1 silencing in Colo205. Despite not being the focus of the current work, we noticed that ABCA1 was recently demonstrated to be a bona fide target of P53 and oncosuppressor in liver cancer^33^. Since P53 was shown to be a target of GLI1 ^34^ and the status of P53 in Colo205 is believed to be wild-type^35^, we speculate that oncogenic GLI1 could indirectly target ABCA1 via P53.

The targeting of HH-GLI pathway in solid tumours has been the focus of previous and current clinical trials involving the targeting of Smoothened (SMO)^36^, however, results have been disappointing so far^37^; this could be ascribed to the frequent activation of HH-GLI by non-canonical oncogenic pathways^5, 7^.

On the other hand, GLI1 has been shown to be a target of arsenic trioxide (ATO), which is FDA approved for the therapy of adult patients with acute promyelocytic leukaemia (APL)^38^. A phase I trial investigating the co-administration of ATO and 5-Fluorouracil/Leucovorin in patients with advanced/relapsed colorectal cancer showed that ATO was well tolerated and that in some patients it was associated with down-regulated thymidylate synthase expression, indicating a therapeutic response, and increased survival^39^; a later study investigated GLI1 levels in biopsies from the said clinical trial and found that it resulted to be down modulated after ATO administration^40^.

In conclusion, the present study provides a rationale for the consideration of HH-GLI pathway as a therapeutic target in CRC patients.

Indeed, our results indicate that the addition of GLI targeting drugs to CRC treatment strategies is a therapeutic option that could prevent the onset of chemoresistance.

## Materials and methods

### Cell culture, treatment, over-expression and drug resistance

Colo205 and HCT15 were obtained from the American Type Culture Collection and were grown in RPMI-1640 (supplemented with 10% (v/v) fetal bovine serum, 1% (v/v) penicillin (50 U ml^−1^)—streptomycin (50 U ml^−1^) and 2 mM l-glutamine). Cells were routinely checked for mycoplasma contamination by testing with PCR Mycoplasma Detection Kit (ABM, Cat. No. G238).

Cells were treated with 10 μM GANT61 (ENZO Lifesciences), and equimolar concentration of Oxaliplatin (Selleckchem) and 5-Fluorouracil (5-FU) (Selleckchem) as described earlier^41^. In detail, to avoid precipitation of the combined Oxaliplatin and 5-FU, we administered 5-FU three hours after Oxaliplatin. For experiments including the combination of GANT61, Oxaliplatin and 5-FU, GANT61 was administered 18 h before Oxaliplatin and 5-FU. For shRNA-mediated knockdown experiments, PLKO lentiviral particles carrying shRNA were purchased from Sigma (1 CFU/cell): MISSION shRNA-non target control Transduction Particles (SCH002V) and three Lenti shGLI1: MISSION shRNA Lentiviral Clone TRCN0000020485, TRCN0000020486 (shGLI1b) and TRCN0000020487 (shGLI1a). Clone TRCN0000020487 demonstrated the best knock-down efficiency with less off target effect and was used for the following experiments^7^. For over-expression experiments, HCT15 were transfected using Lipofectamine 2000 (Invitrogen) according to manufacturer’s instructions.

To induce chemoresistance, cells were treated with increasing and equimolar concentrations of Oxaliplatin and 5-FU. Cells were exposed to a starting concentration of 1 μM in RPMI plus 10% of FBS. The surviving population underwent a stepwise selection of resistant cells by increasing concentration every week to a final concentration of 10 μM.

### Cell viability assay

Cell viability was evaluated by using Celltiter-GLO luminescent cell viability assay (Promega, G7570). CTG reagent was mixed at a 1:1 ratio with supernatant from the treatment plate. The mix was incubated for 10 min at room temperature on the shaker, followed by luminescence measurement using GloMax (Promega).

### Western blot

Cells were lysed in Tris–HCl pH 7.6, 50 mM, deoxycholic acid sodium salt 0.5%, NaCl 140 mM, NP40 1%, EDTA 5 mM, NaF 100 mM, Na pyrophosphate 2 mM and protease inhibitors. Lysates were separated on 8% acrylamide gel and immunoblotted using standard procedures. Primary antibodies were: Anti‐GLI1 (L42B10, Cell Signalling Technology Inc), anti-PARP p85 Fragment (G7341, Promega, Madison USA), anti‐PCNA (D3H8P, Cell Signalling Technology Inc), Anti-ABCA2 (NBP1-20863, Novus Biological), anti-ABCB1 (MDR1, D-11; sc-55510 Santa Cruz Biotechnology, Inc.), anti-ABCB4 (P3II-26, Abcam), anti-ABCB7 (ab151992, Abcam), anti ABCC2 (MDR2, ab3373 M2 III-6, Abcam) anti- ABCG1 (NB400-132, Novus Biological) and anti‐ABCG2 H-70 (sc‐2582; Santa Cruz Biotechnology, Inc.). HRP‐conjugated secondary antisera (Santa Cruz Biotechnology) were used, followed by enhanced chemiluminescence (ECL Amersham, Amersham, UK). Western blots shown in figures are representative of at least three different experiments. Uncropped images for western blots are shown in supplementary Figs. 5 and 6 as indicated in figure legends.

### RNA isolation and real time qPCR

RNA was isolated from cells as previously described^42^. The High Capacity cDNA reverse transcription kit (Applied Biosystems Life Technologies, ThermoFisher) was used to synthesize cDNA. Quantitative reverse transcription (RT-PCR) analysis was performed using a High Capacity cDNA Reverse Transcription kit. mRNA expression was analysed on cDNAs using the ViiA 7 Real-Time PCR System (Life Technologies, SensiFAST Probe Lo-ROX (Bioline), TaqMan gene expression assay according to the manufacturer’s instructions (Life Technologies). Each amplification reaction was performed in triplicate, and the average of the three threshold cycles was used to calculate the amount of transcripts in the sample (SDS software, AB). mRNA quantification was expressed, in arbitrary units, as the ratio of the sample quantity to the calibrator or to the mean values of control samples. All values were normalized to three endogenous controls: HPRT, GAPDH and β-ACTIN.

Primers for gene expression are listed in supplementary Table 1.

Expression of ABC transporter mRNAs was evaluated using the TaqMan Human ABC Transporter Array (Life Technologies).

### Immunofluorescence

Immunofluorescence experiments were performed as previously described^43^ using permanox Labtek chamber slides as support. Briefly, cells were fixed with 4% paraformaldehyde for 10 min at room temperature and permeabilized with 0.1% Triton X-100 in PBS (Sigma-Aldrich, St. Louis, MO). Cells were then blocked with 5% BSA in PBS for 30 min at room temperature and incubated overnight with the following primary antibodies: anti-Gli1 H300 (sc-20687, Santa Cruz Biotechnology Inc.), anti-Gli1 (AF3455, R&D Systems), anti- ABCB1 (MDR1, D-11; sc-55510 Santa Cruz Biotechnology Inc.) and anti‐ABCG2 H-70 (sc‐2582; Santa Cruz Biotechnology, Inc.) diluted in blocking solution. Secondary antibodies conjugated with Alexa Fluor 488 or 594 were purchased from Molecular Probes (Invitrogen) and diluted 1:400 and 1:200, respectively, in blocking solution. Nuclei were Hoechst-counterstained and cover slips were mounted with fluorescence mounting medium (Prolong Gold, Thermo Fisher Scientific, MA, USA). Images were acquired using a FV1200 MPE laser scanning confocal microscope (Olympus) with a UPlanSAPO 20x/0.75 NA objective. Imaris 8.1 software (Oxford Instruments, https://imaris.oxinst.com/) was used for image-processing.

### Chromatin immunoprecipitation (ChIP)

ChIP was performed using the MAGnify Chromatin Immunoprecipitation System (Invitrogen). Protocol was performed as described in^44^. For each ChIP reaction 300,000 cells were used, cell lysates were added to their respective antibody/beads for 2 h. Eluted DNA was PCR amplified with primers encompassing the Gli- responsive elements of human ABC promoter. The following antibodies were used: IgG rabbit (Invitrogen), rabbit polyclonal anti-Gli1 H300 (sc-20687, Santa Cruz Biotechnology Inc.), rabbit polyclonal anti-acetyl-histone 3 (06599, Millipore). Eluted DNA has been analysed with Q-PCR. Primers were designed with Primer-Blast designing tool (https://www.ncbi.nlm.nih.gov/tools/primer-blast/) and Primers tool (Genomatix Genome Analyzer, GGA, v3.30126, https://www.genomatix.de/) and are reported in Supplementary Table 2.

### Datasets and in silico analyses

Datasets available on R2 platform^16^ were interrogated. In detail, the investigated datasets for gene expression were: Disease Colon-Watanabe-121-MAS5.0-u133p2, Watanabe^17^, that investigated gene expression in 53 samples of non-neoplastic rectal mucosa and 67 samples of colorectal cancer; Mixed colorectal cancer microdissected-Galamb-18-MAS5.0-u133p2, Galamb^18^, that investigated gene expression in 6 microdissected samples from colorectal cancer and 6 microdissected samples from non-neoplastic tissues and Tumor Colon CD133+-Carmical-9-MAS5.0-u133p2 public, Carmical^19^, that investigated gene expression in colorectal CD133+ cancer cells and in CAFs.

The following datasets were interrogated for Kaplan Meier analysis, platform used is in brackets: Mixed colon Adenocarcinoma-TCGA-174 (Agilent custom microarray-agg4502a073), that investigated overall survival probability in 174 samples; Tumor Colon (Core-Transcript)-Sveen-333-rma_sketch (Affymetrix Human Exon 1.0 ST array), that investigated event-free survival probability in 333 samples; Tumor Colon CIT (Combat)-Marisa-566-rma (Affymetrix Human Genome U133 Plus 2.0 array), that investigates relapse-free survival probability in 566 samples and Tumor Colon MVRM-SieberSmith-345-fRMA(bc) rma (Affymetrix Human Genome U133 Plus 2.0 array), that investigated relapse free survival probability in 345 samples.

### Statistical analysis

Results are expressed as means + /− SD from an appropriate number of experiments (as indicated in figure legends). Differences were analysed using the Mann–Whitney U-test for non-parametric values, One-way ANOVA and Two-way ANOVA test where appropriate, using the GraphPad Prism software Version 6.0 for macOS, GraphPad Software, San Diego, California USA, https://www.graphpad.com. Adjusted P-values of less than 0.05 were considered as statistically significant.

## Supplementary information

Supplementary Information.
