# Supplementary material for: Hedgehog-GLI signalling promotes chemoresistance through the regulation of ABC transporters in colorectal cancer cells
Source: Sci Rep. 2020 Aug 19;10:13988. doi: 10.1038/s41598-020-70871-9 (PMC7438531; doi:10.1038/s41598-020-70871-9)
Supplement: Supplementary file 1 — Supplementary Information. [file 41598_2020_70871_MOESM1_ESM.pdf]

Hedgehog-GLI signalling promotes chemoresistance through the regulation of ABC transporters in colorectal cancer cells

Agnese Po[1], Anna Citarella[1], Giuseppina Catanzaro[2], Zein Mersini Besharat[2], Sofia Trocchianesi[1], Francesca Gianno[1], Claudia Sabato[2], Marta Moretti[2], Enrico De Smaele[2], Alessandra Vacca[2], Micol Eleonora Fiori[3], Elisabetta Ferretti[2,*]

Affiliations:
[1]Department of Molecular Medicine, Sapienza University of Rome, Viale Regina Elena 291, 00161 Rome, Italy.
[2]Department of Experimental Medicine and Istituto Pasteur Italia - Fondazione Cenci Bolognetti, Sapienza University of Rome, Viale Regina Elena 324, 00161 Rome, Italy.
[3]Department of Oncology and Molecular Medicine, Istituto Superiore di Sanità, 00161 Rome, Italy.

*Correspondence:
Elisabetta Ferretti
elisabetta.ferretti@uniroma1.it

Supplementary Figure 1

GLI1 canonical consensus binding sites: GACCACCCA; CTGGTGGGT
GLI1 non canonical consensus binding sites:: CGCCTCCAG; GCGGAGGTC

ABCA2

```
gctgcgggctggggtgggaagctgatccctcactcactagcccagggtcaccttcctgctgcaaaggagctgggaggagt
ggggagctgtggcctcggaggtcttcattcacttgaggatgcgccttctatgtgcgccctgacactggacaccacacagc
gctactccaccccattcacaggggggaaactgagtctgggaagcgccgtcagagccacccatggggtcagcctgtcctct
gtcgctctctacaggccacgcggaggaactgccccacctcccggggcgccccagcaggtgccaagccacagccgcagaac
cgcctgatcccgaactccctgtgcagagaccagactcgcccgaatatggggagaaaaacgagccgcgtgcagcaggggcc
gcctcggtcccctgcctgctccgctcacggcgagcgttcctggggcctcgagtttccccccttccgctggctgctcggaa
tcgcgggacggacccacctggccgccctgcctccttccaggtgggcaccacctggactccaagtgctggtttacaaagc
acacaggagacccttacgggggtgagctggcccctcgccagcgtccgctcggcgcgccccggggacagtgaaggcttggg
gcgcctctgcagagtcgccagcgcggtgggcgcagccggatgcctctgggagaagagcgcagcgcccgagggtcccccgg
cctacgaaggctaagccggggtctcggattcgatgtgggcgcgtcgcgtgacgcccggcgagggggcggcccggaggcttc
ccggcggcggcgcgcgcagggtctcgggcccgggtcggtcccgggcgcggcgcgggcggcgggcgcctcctggcgggcgc
ggacggagcgcggcgcctttaagcgggggcgggcggggcgcgggcagagtcgctgtcgccgcggccgccggggcggagcc
agcgcggatcgggtcccggacgcccgagcgccccgcccccGCGCGGGCGATGCCCAGCGGCGCGGCGGGCTGCGGGGCCC
GGCGGGGCGCGCAGAGGAGCGGGCCGCGGCGCTGAGGCGGCGGAGCGTGGCCCCGCC
```

ABCB1

```
tgtgtgtctattatttctcaacctgccgatccgcctaagaacaaagagagagccccgttgcattgcaggctgctggccag
atcccacaatacatacacagattccaaaatgcattcttaactcttaaaagattttggttacttatcactattctgtctac
ttttctgtaattgagaaaagtatttgtcaactcatttttctctctgtgacagctcagtcatttacaaagttttatttta
tactttactccttccttcaatttgtgctaaaacattgtgaaaattaacatttctttggaacacaacttttttcattataaa
ttaaatactgatataattgcaaagtaaacaaatgaatttccataaagctaatttatctttatattttccatacttattac
ttcaaattcttgttacattttcaattttgtttgaaatatcatatggtattttaaattgaaatttactaattattttttag
ccagtggataaagagaaattaaaaatttgaacaaattaattcaatttttacttcacttctcatttgaaggtcttcccagt
aacctaccaaaagaagtctctctcttttttttttttttttttagcttagattctaatcttcattctgcctattctggcta
aacttctcaactctggctatttttcaattataccccaatccctaagccatgtaactcttcgaggtttttgtttgtttttt
caactgctcattaagacgtcctacaccttagcaaaagatcacacatatttcttcaatgctttggagccatagtcatgta
ctcaaaatttattttatctctactcccacccttcctccacccaaacttatccttgggttttcactgattagtgctttcaa
gcctgcctgccttagttcatgtagctcctcctctggtactgggataaacacttgtattaccattttaaaggctatcatta
ctctttacctgtgaagagtagaacatgaagaaatctacttACTCTGACTGTATGAGATGTTAAATACTTTTTAATATTTG
TTTAGATATGACATTTATTCAAAGTTAAAAGCAAACACTTACAGAATTATGAAGAGGTATCTGTTTAACATTTCCTCAGT
CAAGTTCAGAGTCTTCAGAGACTTCGTAATTAAAGGAACAGAGTGAGAGACATCATCAAGTGGAGAGAAATCATAGTTTA
```

ABCB4

```
cctcgcaataacctgataagggagatgtttttatcctcattttacatataaggaaacaggcctagagaaatgagcacagt
gtccaaagtcacatagttaataagatgtgaagctctgagtttgaaagtctccggtttcaaagccatgaaacttatggctc
cccgttttagacacttcctttgggaagagtgtggaggaattaatcagaaagaagaaagtcatactcaaataggtggtag
gagcagagacaattcaatacagacagaagtcttagatgagagcagtgagccagggcactggactgggactcaggaggctt
cccctagactctggttccaccgatgcagcctcaggcaggacttcacctctctgggcatccgtttcttcatatgttaaaca
tacggggttttaattagatgatcgctgaagaccctctagccctaaaactctgtgtctcttaagtgctaagagggcacca
acagcgttcctcctccccaaggagcataatgtgatggttcctgccggccctggctgactctcgccgtccttggagataat
tgggttcagtgccacctggaccagaactggggatgcggaagcaagaggcgagtctattgctctctctcggtcctgggccg
ccctgtgattgttgggcgtccggaaactgtctcccctatgggtttaaaaacaaaactgagcgcccatggggtgtgacagt
catctgcaggggcttgggtggcccatcaggcgaggctttctcggcacccgaggctccagcctgatctcggtcttatcctg
cgaccgggctggttctggcgggtcgccagggtgggcggcggccccagccgggcgccccggcggcaagagcggcaggctgc
gcccctggcccgcgcctagcctggggagagagctgggcgggcggcgggagctgctctcgcgggccgcggccctcgccctg
gctgcaacggtaggcgtttcccgggccggacgcgcgtgggGGCGGGGGCGGGGGCGGGGGCGAGGCCGCGGCGAGCAAA
GTCCAGGCCCCTCTGCTGCAGCGCCCGCGCGTCCAGAGGCCCTGCCAGACACGCGCGAGGTTCGAGGCTGAG
```

Supplementary Figure 1 (continued)

ABCB7

caaagtacctcttcataagaagtccataattatttgtctcttttgtgtgaaaagttggagaatgcagcagtgaatataaa
attgcaactgcattattgtctccggggtttcaggtaacatttacaactggaaatgcagtttcctagataactgtaaacat
attttgacattttgtttgataatagtggtgacaaaagaatataagcctgtctttaaacatggtcttctctccatacattt
cactttaccacccttcagccaacctaagcgatttttcaagaattagggacgctgcaaaaaccaaagcgaagagctagaaa
gagtgacaaaattagtaaagatgtctgtagtctctctgtgtgtgtgtgtgtgtgtgtgtgtgtgagactgtcccacgagc
aacagaccaaaggaaaactgccccaaggtttgcgcaacagagaacaatcagcaaagttaaagtccttacaggacagaaaa
gcagtaaatcttacagctgaagcctcctcccaggggaaattaacgtctgtagtttaatctttccttaattcagaagggag
tgaagataagaatatgcatagggaaaatggagagaggggagcgtgacattgagttggaggaggtaagagagtattatgag
agacaagatggggttgacagatttagcagatacaatatttgggacacacgcacgaagagatgattcgttgtttatgtaaa
attgaaacttaactaggtaggcatcctgtatttcatctggcaactccaggaaaagagaaaaatacagtgttactccaata
ttcccggttgtgtaaggaaaacaatcaaagctcggccctaaggaaaaacaaataaaagaggctgtgcgaatgcatccgaa
tcttcaaacagaatccatttccggtctggggccctgggaaaacaagaagaggagagacaattaaggtagccgaattcagt
ccgccagtgtcccataatcctcttctctcggttcctctttCCTCGCTCAAG

ABCC2

ccaattttttttagctaggatactgcatgggtggttatgttttagctaggataccgcatgggtggttcatgatatcatga
aaaaagcatgttcttaaagcaatttaagtgacagtacaaaaggttgggtcaggtgggcacggtagctcatgcctgcaat
cccagccctttgggaggccaaggcagaaggattgttgaagcctggagtttgagaccagcctgggcaacatagtgagaccc
cgtccctacagaaaacatttttttaattagctgggcttgttggcatgtgcctgtagtcccagctactcaggaagctgagg
caggaagatcgcttgaacccatgaggtcaaggctgcaatgaatcatgatggcaacactgcactctagctcaggcaacaga
ccaagaccctgtctcaaaaaaacaaaaaacaataacaaaaaaagaaaaggttgggtcgatgacagtttctagcgactgatg
ccaccactctgtttacagaatcaccccctctcctctggccctgatgttttatcatgatatcatatttacaatgcctggcaa
aggcttcattcccatttggcatactacctctcaggcaaatagaacttttgaaagcctgtattatgtatataacatatagg
ctcacactggataagctattttataacctgacttcttcaaagaaagtttacatcatgtttaaaccatgttttagattcta
tatttttaattaaaaatctaaggaagaaggatatttcacatttctataaactctaagatcttgcagcagaagcgaaactg
cacatttaggggtgcctgcccctctactgatgctgccctttgtgggtcatatgtccttaggaaaatgaaagactgtgcac
tcttgatttgttggccagctctgttgacatctttcagtggttccttttatgtatggccactcctacagaggcctcttgta
ctttgggaactggtgagtctccctgtccctagggctttttAGTCACATGTCCATCCACTGTTTCAATGTAACATGCATCT
AGGCAAGGTTAACGATTAAATGGTTGGGATGAAAGGTCATCCTTTACGGAGAACATCAGAATGGTAGATAATTCCTGTTC
CACTTTCTTTGATGAAACAAGTAAAGAAGAAACAACACAATCATATTAATAGAAGAGTCTTCGTTCCAGACGCAGTCCAG
GAATC

ABCG1

aaaagctgtgcctcctttctaggtgaggagaaagagagagacctggctcatctgaggtgtggttgggaggggggacccag
gtgtgctggaaatgaaaagaaatgcattcctgtttttcgtcccaacatgcaaacaactgaacaaaagcattagggcctga
gactgggagtaaagaattccttgtcaccatggataccaggaaatggccccacttatatataataagggctttagagatgc
tggaccatctgatattccagcctggggccacatgggagtgtgccctggtgttattccttatacagttccatgaacatggc
tctggaaacacctctgtctgcagaaaatgaggcttttctttttttgttcgggggtgaacagagggcagaggcctgggcat
cttcactcagcacccctttgtaacccagcacttagcaccatggctggcgcacagcaatgtcacatgtgtgagtgcacacg
atgcctcactgccaggggtcaccccacaccggtgctgttgggggcgttggagtggttatctcttctttagtcctcaagct
cctacctggcagagagctgcccaacaccgtcggggtggggtgggcgggaagggaagaagcagcagcaagaaagaagcccc
ctggccctcactctccctccctggacgccccctcttcgaccccatcacacagccgcttgaggcttggaggcagtggattt
ccgagcctgggaaccccggcgtctgtccggtgtccccgcagcctcacccgtgctggcccagcccccgcgagttcgggacc
cggggtttccggggtggcagggggttcccatgccgcctgcgaggcctcggctcgggccgctcccggaacctgcacttcag
gggtcctggtccgccgcccccagcaggagcaaaacaagagcacgcgcacctgccggcccgcccgccccccttggtgccggc
caatcgcgcgctcggggcggggtcgggcgcgctggaaccaGAGCCGGAGCCGGATCCCAGCCGGAGCCCAAGCGCAGCCC
GCACCCCGCGCAGCGGCTGAGCCGGGAGCCAGCGCAGCCTCGGCCCCGCAGCTCAAGCCTCGTCCCCGCCGCCGCCGCCG
CCGCCGCCGCCGCCGCCCCCGGGGC

Supplementary Figure 2

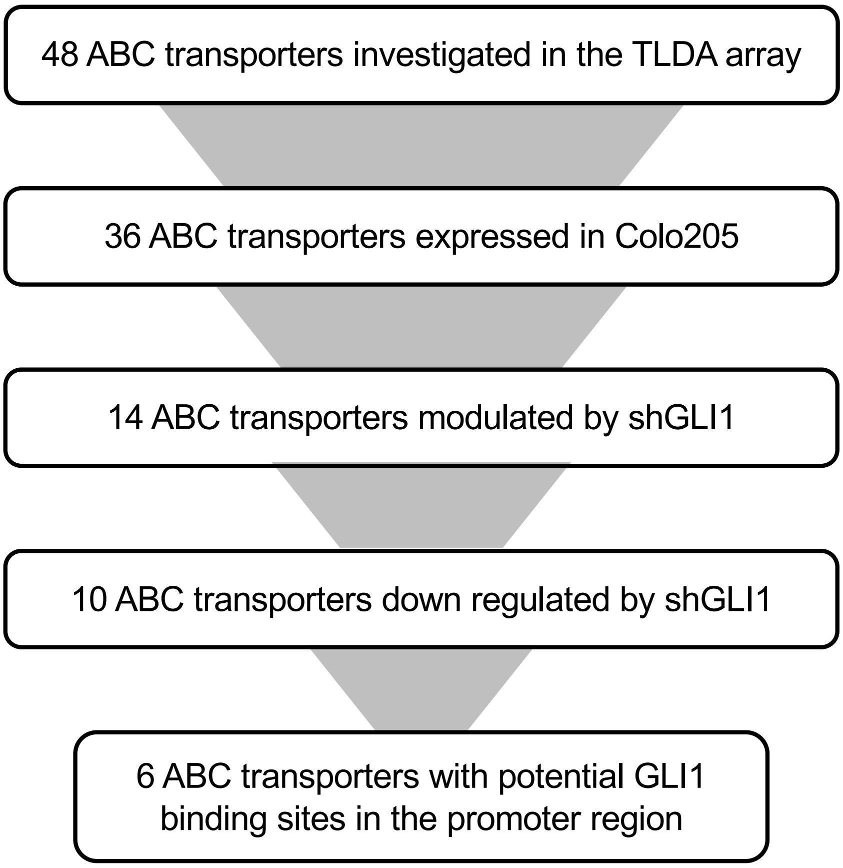

Supplementary Figure 3

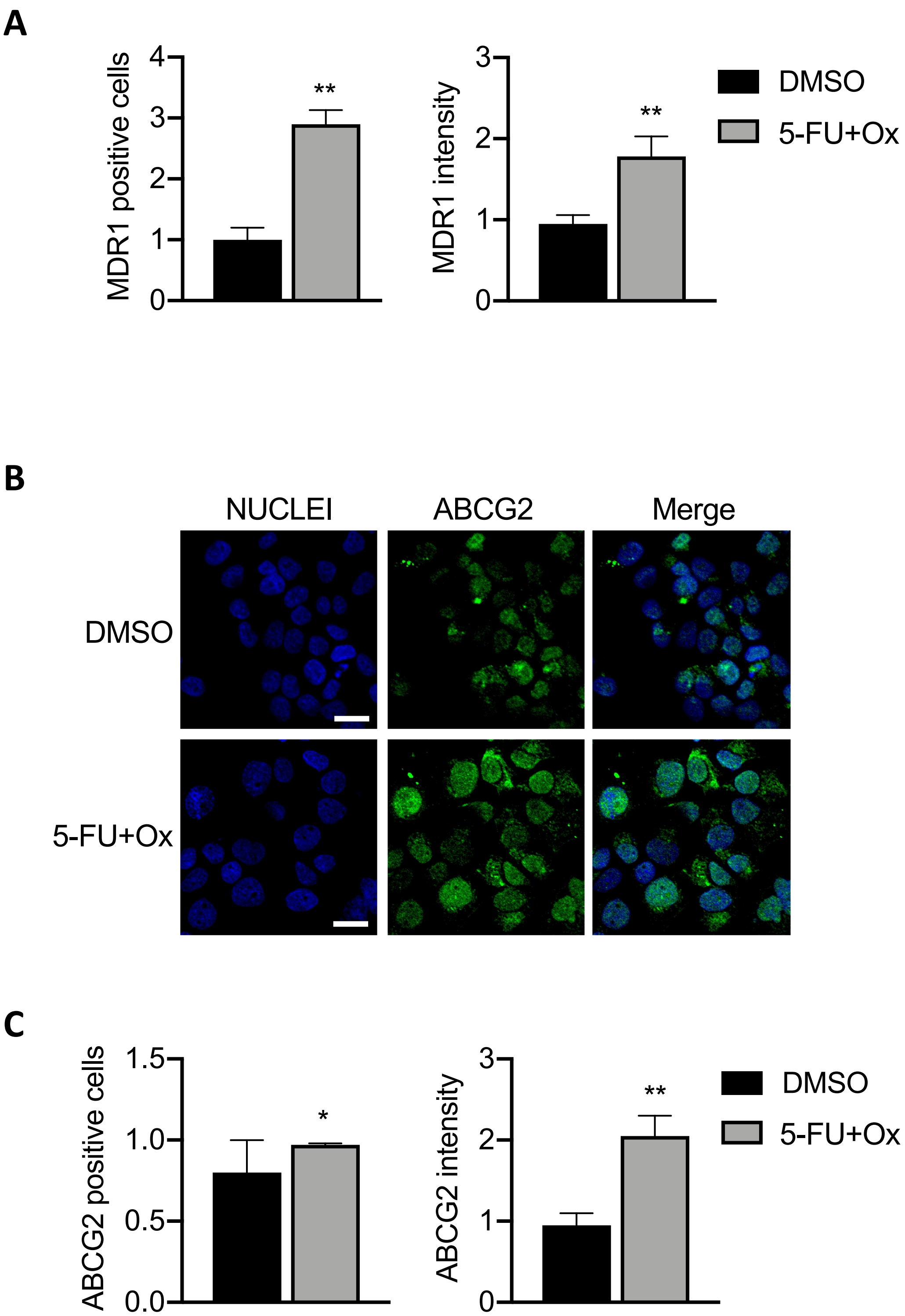

Supplementary Figure 4

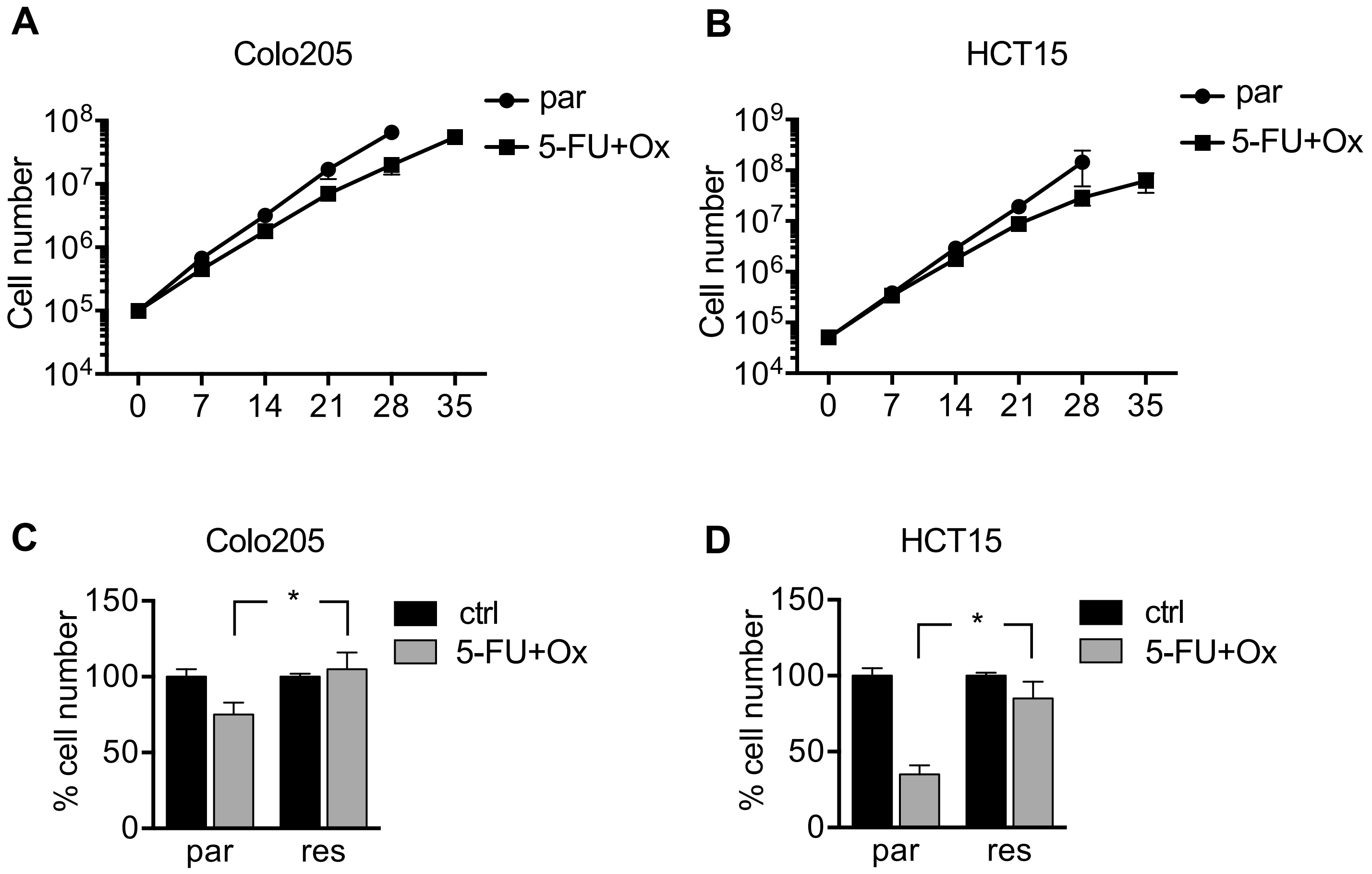

Supplementary Figure 5

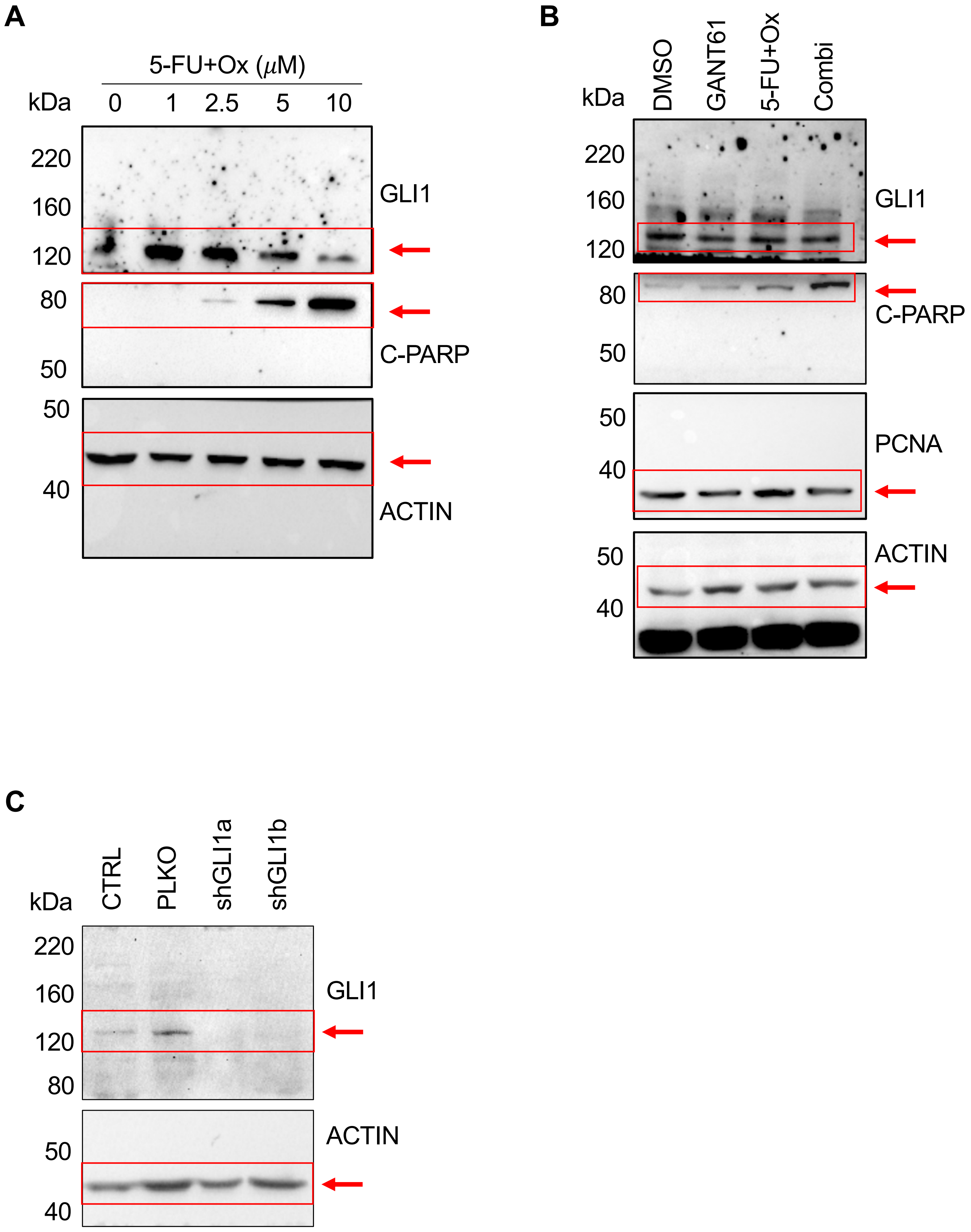

Supplementary Figure 6

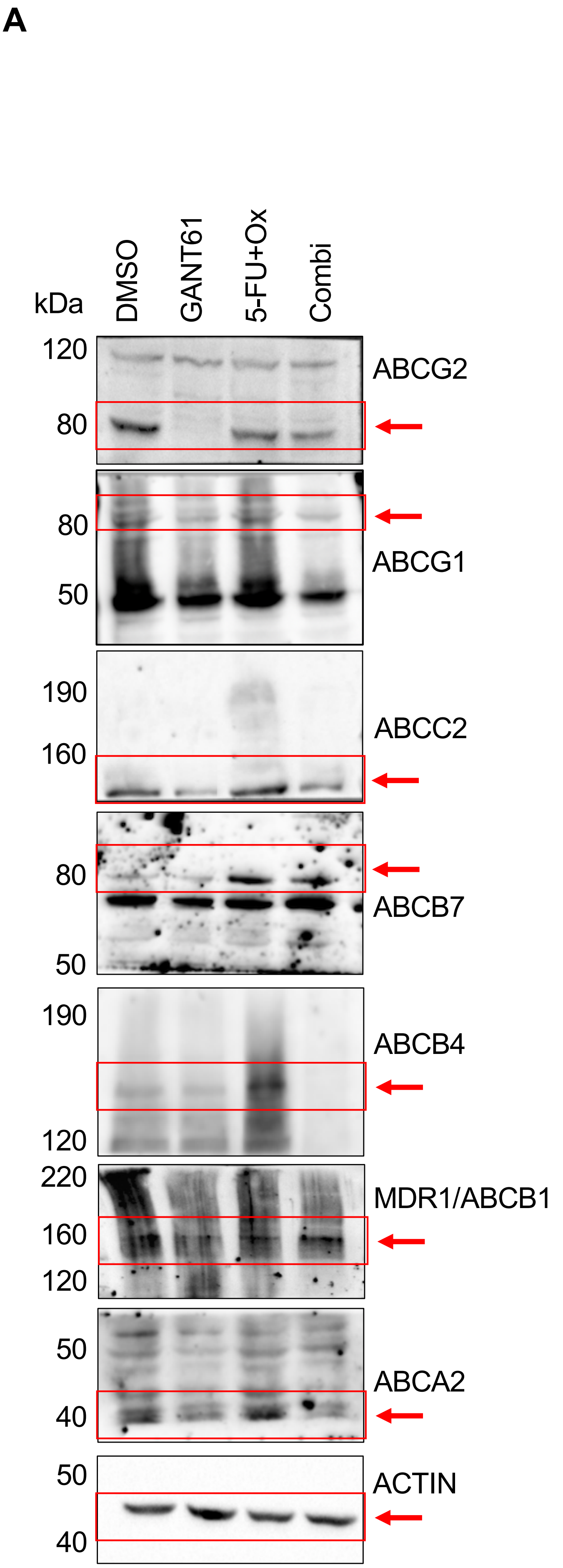

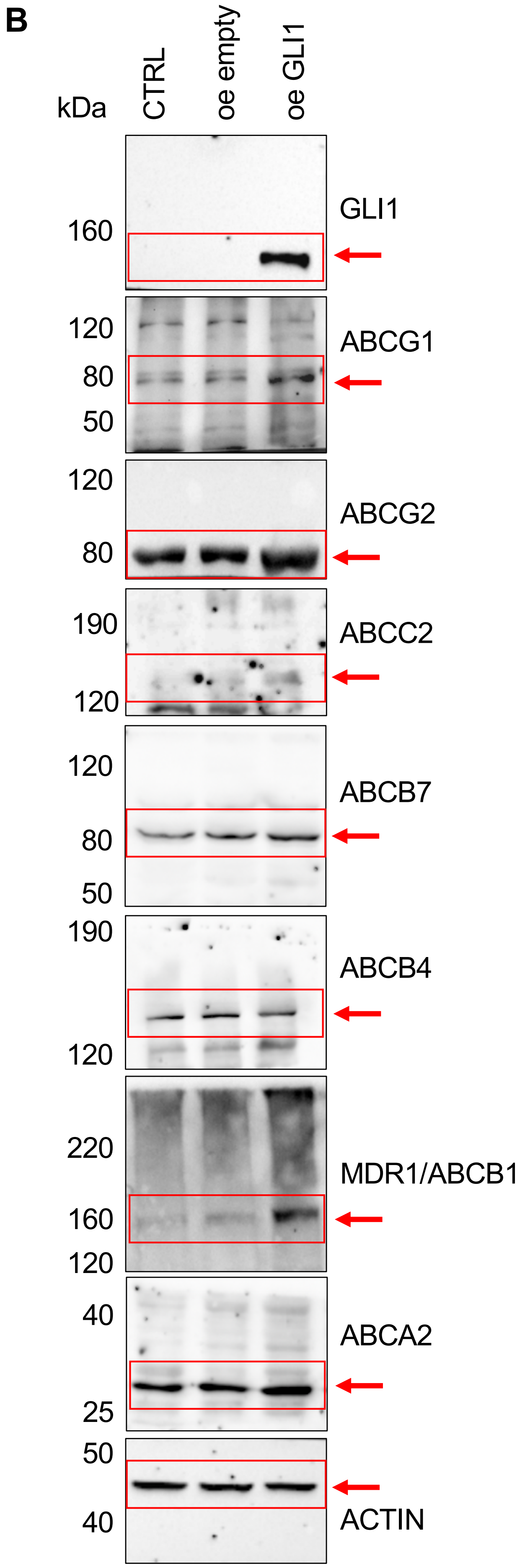

Supplementary Table 1

	FORWARD	REVERSE
GLI1	GCAGACACACTGGCGAGAAG	AGGTTTTCGAGGCGTGAGTA
ABCA2	TGGAAGAACGTGACGCTCA	ACGGAGATGGTGGGCTTCTT
ABCB1	AGGAAGCAACCAGATAAAAGATCAA	CACCTTCTTGTCCTCCGCTC
ABCB4	GGACAGTGCTTCTCGATGGT	CCGGCTGTTGTCTCCATAGG
ABCB7	GCCTACCAGCAGATTCCAGAG	GTCCTGAATTGCCTTTTCCCA
ABCC2	TTCTGCAACTCTACTTTTTGGAATT	CCACACCAGAACAGTTTGCT
ABCG1	AGGCAGAAGGGAAATGGTCAA	AGAACATGACTGGAGGGTTGT
ABCG2	TCAGCTGGTTATCACTGTGAGG	GGCTCTATGATCTCTGTGGCT

Supplementary table 1 - List of primers for gene expression

Supplementary Table 2

	FORWARD	REVERSE
ABCA2	CAAAGGAGCTGGGAGGAGTG	TGTCAGGGCGCACATAGAAG
ABCB1	TCTCTACTCCCACCCTTCCTC	AAGGCAGGCAGGCTTGAAA
ABCB4	CTCGCCGTCCTTGGAGATAA	GGGAGACAGTTTCCGGACG
ABCB7	TTACCACCCTTCAGCCAACC	GCTCTTCGCTTTGGTTTTTGC
ABCC2	ACTGCATGGGTGGTTATGTTTTAG	CCTGACCCAACCTTTTGTACTG
ABCG1	CTGGGCATCTTCACTCAGCA	AGATAACCACTCCAACGCCC
ABCG2	TGCTGTGCCCACTCAAAAGG	CTTGCCGCGTCTCTCAATCT

Supplementary table 2 - List of primers used for ChIP experiments

Supplementary figure legends

Supplementary figure 1. Promoter region of the selected ABC transporters were analysed for the presence of potential GLI1 binding sites. Consensus binding sites are highlighted.

Supplementary figure 2. Scheme summarizing the filtering process to select ABC transporters for the experiments shown in figures 3, 4, 5 and 6.

Supplementary figure 3. (A) Right: Histogram showing MDR1/ABCB1 positive cells in Colo205 treated with 10μM 5-FU and Oxaliplatin (5-FU+Ox), quantified as the ratio between MDR1/ABCB1 positive cells and the total number of cells; Left: Histogram showing MDR1/ABCB1 intensity, evaluated as the ratio between MDR1/ABCB1 intensity and Hoechst intensity. ** $p<0,001$ in 5-FU+Ox vs DMSO treated cells (two-tailed unpaired t-test) (B) Immunofluorescence staining of ABCG2 expression in Colo205 cells after 5-FU+Ox treatment or control (DMSO). Bars, 20 μm. Images are representative of at least three independent experiments. (C) Right: Histogram showing ABCG2 positive cells in Colo205 treated with 10μM 5-FU+Ox, quantified as the ratio between ABCG2 positive cells and the total number of cells; Left: Histogram showing ABCG2 intensity, evaluated as the ratio between ABCG2 intensity and Hoechst intensity. *$p<0.05$, ** $p<0.001$ in 5-FU+Ox vs DMSO treated cells (Unpaired t-test).

Supplementary figure 4. Cell growth measured in (A) Colo205 and (B) HCT15 in parental population (par) and during chronic exposure to 5-FU and Oxaliplatin (5-FU+Ox) (One-way ANOVA test). Cells were treated with increasing doses 5-FU+Ox, starting at 1μM at day 0 and increasing the concentration weekly up to 10μM during the indicated timespan. Cell growth measured in (C) Colo205 and (D) HCT15 in parental (par) and resistant (res) cells exposed to 10μM 5-FU+Ox for 48 hours. *$p<0.05$ in resistant versus parental 5-FU+Ox treated cells (Two-way ANOVA test).

Supplementary figure 5. Raw western blot images for images found in main figures. Red boxes and arrow indicate cropped areas. (A) uncropped images for Figure 1A (B) uncropped images for Figure 1D (C) uncropped images for Figure 2A.

Supplementary figure 6. Raw western blot images for images found in main figures. Red boxes and arrow indicate cropped areas. (A) uncropped images for Figure 4B (B) uncropped images for Figure 4E.

Supplementary tables

Supplementary table 1.
List of primers used for gene expression.

Supplementary table 2.
List of primers used for ChIP experiments.
